# The predominant stride-frequency for routine swimming in catsharks (*Scyliorhinus canicula*) generates high power at high efficiency in the red musculature

**DOI:** 10.1007/s10974-022-09637-x

**Published:** 2022-11-23

**Authors:** Timothy G. West, Nancy A. Curtin, Roger C. Woledge

**Affiliations:** 1 Structure & Motion Laboratory, Royal Veterinary College, University of London, Hawkshead Lane, Hatfield, AL9 7TA UK; 2 Cardio-Respiratory Interface, NHLI, Imperial College London, London, SW7 2AZ UK

**Keywords:** Shark swimming, Red fibres, Power, Efficiency, Stride-frequency

## Abstract

Videos of free swimming of catsharks (*Scyliorhinus canicula*) were analysed to give values of swimming speed (units: FL (fish lengths) s^−1^), stride-length (forward movement in the direction of travel per cycle of body undulation (units: FL) and stride-frequency (units: s^−1^). Most of the swims (139 of 163, 85%) were at speeds less than 0.545 FL s^−1^ and were categorized as slow. The rest (24/163, 15%) were categorized as fast. Stride-lengths and stride-frequencies could be evaluated for 115 of the slow swims and 16 of the fast swims. We discuss the fast swim results, but there were so few fast swims that no firm conclusions could be made. As swim speed increased during slow swims, there was a strong increase stride-length [slope 0.965, P < 0.0001)] and a small increase in stride-frequency. Most stride-frequencies (70/115, 61%) were in the range 0.68–0.88 s^−1^. Previous experiments on red muscle isolated of catshark showed that in this range of frequencies of sinusoidal movement, high power was produced at high efficiency (Curtin and Woledge b). Lower frequencies gave less power and at higher frequencies the efficiency of energy conversion was lower. Thus, we conclude that during routine swimming catsharks choose a swimming speed that optimizes red muscle performance in terms of power and efficiency.

**Supplementary Information:**

The online version contains supplementary material available at 10.1007/s10974-022-09637-x.

## Introduction

The investigation of the properties of different types of muscle from various animal species has a long and continuing history (Needham 1971; Squire 1981). John Squire’s interest in the structure of muscle from a huge range of animals, both vertebrates and invertebrates, included the ultrastructure of the red and white fibres of catshark (*Scyliorhinus canicula*). These fibres were used in physiological experiments relevant to this report and lead to our first direct contact with John and his group, when we provided Pradeep Luther with catshark fibres for a study of the myosin arrangement across species. The myosin filament has a triangular profile in cross-section. In simple lattice muscles all the triangles orient in the same direction, and in superlattice muscles the triangles orient in one of two directions. Luther et al (1996) showed that catshark muscle is unusual in containing both the simple lattice form in red fibres and super-lattice form in white fibres. Thus, started continuing contact and friendship.

Catshark muscle is particularly suitable for studies of red slow-twitch fibres and white fast-twitch fibres because they are segregated in different parts of the body. The red fibres are in a relative thin band just under the skin with their long axis aligned along the length of the body. The white fibres make up the rest of the body musculature (myotomal muscle) and are arranged in a complex conical pattern. In addition, the colour contrast is extreme compared with the corresponding fibres in mammalian muscle. Thus, a preparation containing a single fibre type can be prepared relatively easily from catshark.

Catshark swimming is anguilliform, or eel-like, in that a wave of sinusoidal undulation travels from head to tail along the body (Gray 1933, 1968) and provides the main propulsive force. Observations on spinal catshark (spinal cord severed caudal to the brain) show that muscle fibres are activated during part of each sinusoidal cycle. EMG activity occurs in red, but not white fibres, during the slow swimming movements of spinal catshark, and white fibres become active during vigorous movement (Bone 1966). Similar observations have been made of motor neurone activity, including action potential frequency, to red fibres (Mos et al. 1990). Guided by these findings about the activation patterns, we have previously used fibres isolated from the catshark (in vitro experiments) to characterize the mechanical and energetic performance of the red and white fibre types (Curtin and Woledge 1993a, b; Curtin et al. 1998, 2005, 2010; West et al. 2004, and references therein). The maximum power output of red fibres is less and is produced at a lower movement frequency than that of white fibres during optimized sinusoidal movement and stimulation patterns. The results also show the red fibres convert energy more efficiently than white fibres (Curtin and Woledge 1993a, b). How do these measurements of muscle fibres contracting in vitro compare with the performance of the fibre types in live swimming catshark? Here we report some answers based on analysis of video recordings of catshark swimming during routine swimming in a large tank of still water.

## Methods

*Scyliorhinus canicula* (small spotted catshark, also known as dogfish) were videoed from above while spontaneously swimming in a large circular tank at the Aberdeen Marine Laboratory. The water temperature was 12 °C. Video footage (30 fps) was digitised into MGP and WVM formats and these were cut into 20, 30 or 60 s files for analysis of swim-paths in MatLab (The MathWorks, Inc, MA, USA). Several fish will have been captured in a single video; every fish that swam through the swim arena could be analysed and a separate swim path saved for each. A 1 m reference pattern was visible in each video, providing a calibration for all fish sizes and movements of 181.8182 pixel m^−1^.

Individual fish were followed after first loading a video clip into a MatLab tracking protocol, then marking the snout of a fish at the beginning of its swim, and finally tracing the snout movement in an x–y (pixel) swim-path. At the end of a swim, the video playback and tracking stopped automatically, and the swim path was saved. A series of markers was placed on the still image of the fish, from snout to tail and closely following the curvature of the fish. Fish length (FL) was then determined as the summation of the short linear segments between the markers.

**Fig. 1:**
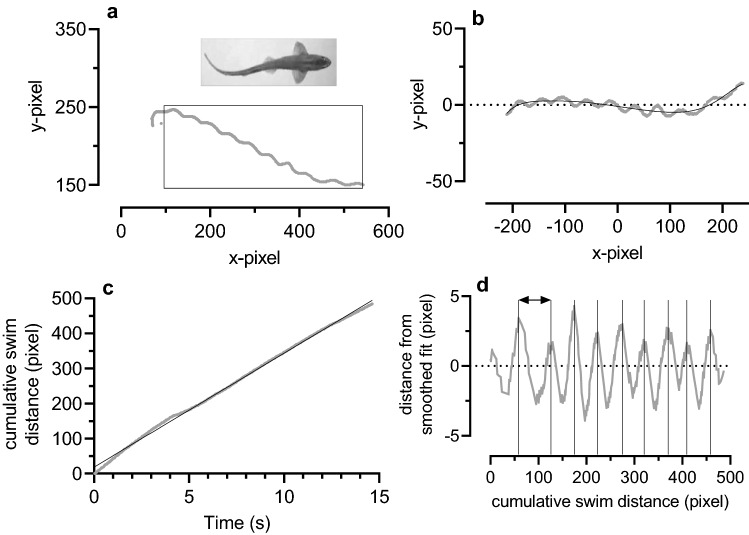
An illustration of swim path analysis for a 0.58 m long catshark **a** Recording of the swim path was begun by loading a digitised video recording into a MatLab tracking script and marking the snout of a selected fish in the first frame. As the recording proceeded, the position of the snout was tracked and x, y pixel values were collected at the film frame rate (30 fps). A region of interest was identified on the swim-path (black box around the grey swim path). **b** The region of interest was rotated so that the direction of swim was as close as possible to horizontal. The point x = 0 and y = 0 in *Panel b* corresponds to the midpoints of x and y in the box in *Panel a*. A polynomial, in this case an 8^th^ degree polynomial, generated a smoothed swim path (black line through the grey data points). **c** Simple linear regression of the relationship between cumulative swim distance (along the non-smoothed path) and the swim time (starting at 1/30th s) gave a swim speed of 32.9 pixel s^−1^ (0.18 m s^−1^, or 0.31 FL s^−1^). **d** The difference in distance-travelled along the original and the smoothed paths shown in *Panel b* were plotted against cumulative distance moved in the smoothed path. The peaks were identified manually and marked by the vertical lines. The distances between successive pairs of peaks (for example the horizontal double headed arrow) are the stride lengths (in pixels) during the swim. In this example, stride length ranged from 39.9 to 64.3 pixels (0.22–0.35 m, or 0.38–0.61 FL) and averaged 47.0 pixels. Stride-frequency (swim speed / average stride length) was 0.7 s^−1^

Further analysis of the swim path is illustrated in Fig. 1. First, a region of interest was identified on each swim-path. In cases where the swim-path was largely in one direction, the region of interest could be chosen as nearly the entire swim (as in Fig. 1a). Swim paths with a clear change in path-direction were analysed as two separate swims. The region of interest was rotated to maximise distance in the x-domain and then re-plotted with the origin as x–y midpoints of the swim-path region of interest (Fig. 1b). A multidimensional (up to 10th degree) polynomial curve-fit generated a smoothed swim-path (Fig. 1b). The Pythagorean Theorem was used to determine point-by-point segment lengths along both of these paths. Distances travelled along each path were the cumulative sum of the segment lengths. The slope of a linear fit to the relationship between distance travelled in the non-smoothed path and swim time (from the camera frame rate of 30 fps) provided an estimate of average speed during the swim (Fig. 1c). The difference between the non-smoothed and smoothed distances travelled, plotted against the distance travelled in the smoothed path (Fig. 1d), depicts the number of strides and the stride-lengths taken by the fish during the swim. Average stride length and stride frequency (swim speed divided by stride length) were recorded for each swim path.

**Fig. 2:**
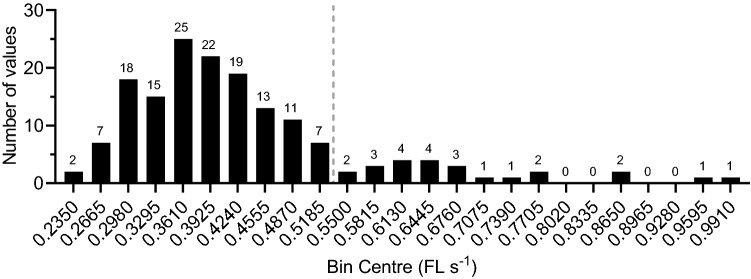
Frequency distribution of catshark swim speeds. Bin-centres are speeds in FL s^−1^; each bin-width is 0.0315 FL s^−1^. Of the total 163 measurements, 139 (85%) were classed as slow swims and 24 (15%) as fast swims. The vertical broken line separates the fast and slow groupings; the upper limit of slow swims was just over one-half FL s^−1^ (0.5185 + (0.0315/2) = 0.5343 FL s^−1^). The fast swims occurred over a wide range of speeds, 0.5343–1.0068 FL s^−1^, a clear preferred range of fast speeds was not evident

A total of 163 swims were analysed, for fish ranging 0.463–0.706 m in length. These were classified as fast speed or slow speed swims, based on the frequency distribution of swim speed (see results, Fig. 2). Of these, 115 of the slow swims, and 16 of the fast swims, yielded stride data. Frequency distributions and simple linear regression analyses were used to evaluate the relationships between stride-length and stride-frequency as a function of swim speed. Speed and stride data have been expressed relative to body-length; average (± SEM). Speeds are presented as fish-lengths (FL) s^−1^ and strides in FL. GraphPad Prism V 9.0.0 (GraphPad Software, San Deigo, Ca USA) was used for histogram and regression analyses.

## Results

The histogram of swim speeds (Fig. 2) shows that most swims were at speeds at the lower end of the range of speeds we observed. There were several swims scattered over a wide range of higher speeds. The swims were divided into two groups on the basis on speed; speeds less than 0.535 FL s^−1^ were categorized as “slow” (139 of the 163 total, 85% of the swims) and those at greater speeds were “fast” (24, or 15% of the total). The slow swims occurred in the speed range, 0.178–0.534 FL s^−1^, with a symmetrical distribution and median speed, 0.381 FL s^−1^. The fast swims occurred over a wider speed range, 0.535–1.007 FL s^−1^, and a clear median or preferred speed was not evident.

As described in the Methods, some swims (115 slow swims, and 16 fast swims) could be analysed to give values for the stride-length (units: FL), the distance moved in the forward direction during one cycle of side-to-side movement of the fish body. The stride frequency (s^−1^) was calculated as swim speed (units: FL s^−1^) divided by stride length (units: FL). In anguilliform-like swimmers, including catshark, stride-frequency corresponds to the frequency of the lateral movements of the snout (Gray 1933), the undulations of the fish body and tail beat, though the amplitudes of lateral movement varies.

**Fig. 3:**
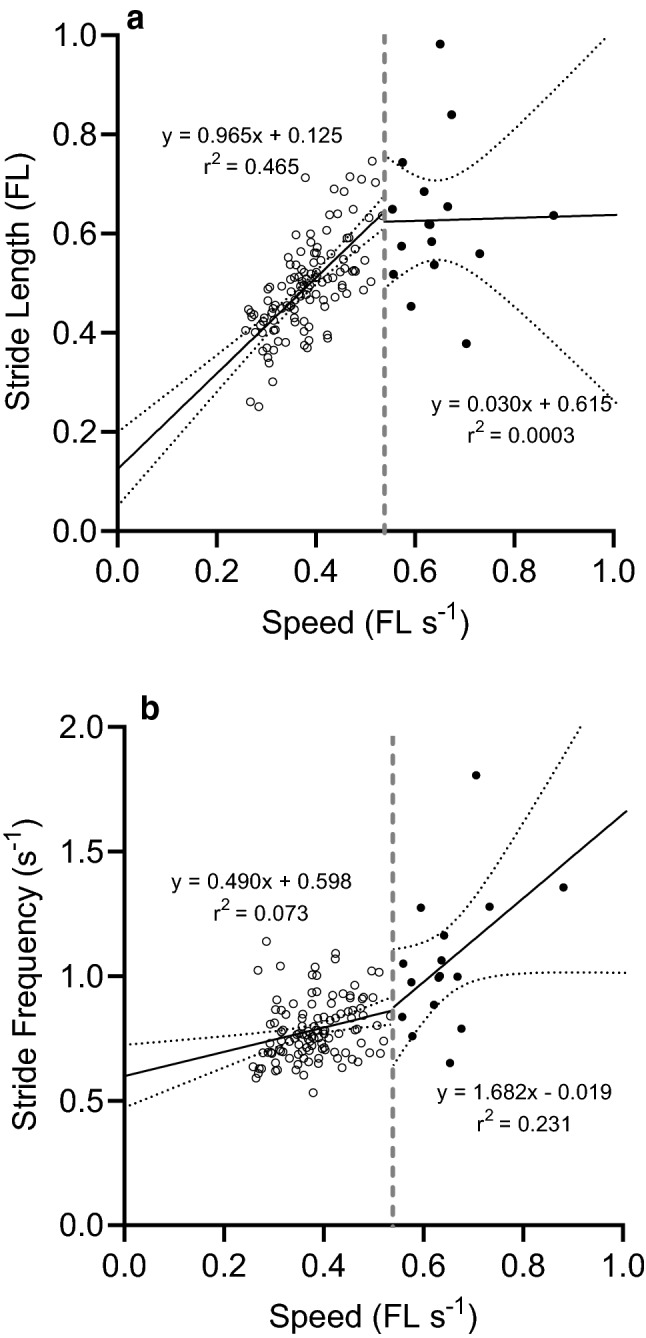
Relation between catshark swim speed and stride-length and frequency. Regression analysis of the dependence of catshark slow (empty circle) and fast (solid circle) swim speed on stride-length (*Panel a*) and stride-frequency (*Panel b*). Simple linear regression lines (black) with 95% CI (confidence intervals) are depicted. The vertical broken lines separate the stride-lengths and stride-frequencies determined for the slow and fast swim speeds. **a** Stride-length explained 46.5% of the variance seen in the slow swim speeds (slope is significantly non-zero; F = 98.3 with 1,113 d.f.; P < 0.0001), but essentially none of the variance in fast swim speed (F = 0.0038 with 1, 14 d.f.; P = 0.952). **b** Little of the variance in slow swim speed was explained by stride-frequency, but the regression slope is significantly different from zero (F = 8.88 with 1, 113 df; P = 0.0035). The slope of the relationship between stride-frequency and fast swim speed was marginally non-significant (F = 4.21 with 1,14 d.f.; P = 0.059)

**Fig. 4:**
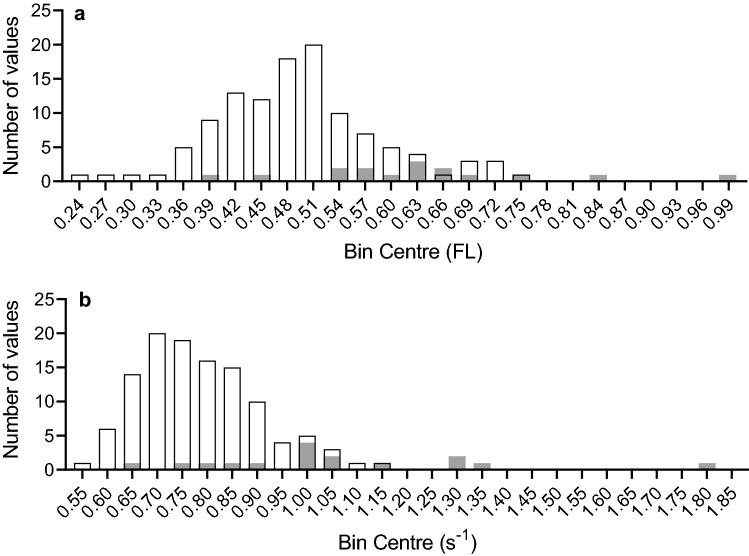
Frequency distribution of stride-length and stride-frequency during catshark swims. Of the 163 swims analysed for speed (see Fig. 2), 131 also yielded data for stride-length and stride-frequency. White bars are values for slow swims (n = 115) and Gray bars for fast swims (n = 16). **A** Bin centres for the distribution of stride-lengths are in units FL, and bin widths are 0.03 FL. Average stride-length for the slow swims was 0.49 ± 0.009 (SEM) FL and the median stride-length was also 0.49 FL. The range of slow strides determined was 0.25–0.75 FL. Stride-lengths for the 16 faster swims occurred over FL of 0.39–0.99; average fast stride-length was 0.63 ± 0.036 (SEM) FL, the median was also 0.63 FL. **B** Bin centres for the distribution of stride-frequencies are in units s^−1^, and bin widths are 0.05 s^−1^. Average stride-frequency for the slow swims was 0.78 ± 0.011 s^−1^; median slow stride-frequency was 0.77 s^−1^. The range of slow stride-frequencies was from 0.53 to 1.14 s^−1^. Stride-frequencies for the 16 faster swims were in the range 0.66–1.805 s^−1^. Average fast stride-frequency was 1.06 ± 0.070 s^−1^; the median fast stride-frequency was 1.004 s^−1^

Figure 3 shows the clear relationships between changes in slow swim speed and (a) the average stride-length during a swim and (b) stride-frequency during a swim. During slow swimming, stride-length increased as speed increased (Fig. 3a), with a slight, but statistically significant, increase in stride-frequency (Fig. 3b). There are relatively few results for fast swimming; these speeds were chosen by the catshark rather than speeds due to control imposed by the experimenter. Although sparse, the available results suggest that during fast swimming stride-length levelled off as speed increased. In contrast, stride-frequency increased as speed increased and increased more steeply than during slow swims. Figure 4 shows the histograms of (a) stride-length and (b) stride-frequency.

## Discussion

Our main aim was to compare the observations of free-swimming catshark reported here with in vitro isolated muscle fibre measurements of power and efficiency. For the in vitro experiments the muscle fibre bundles were stimulated during sinusoidal movement and power was evaluated by the work loop technique (Josephson 1985, 1993).

Efficiency as defined here is a measure of the fraction of energy from concurrent chemical reactions in the muscle fibres that is converted into mechanical power. Efficiency is evaluated as the power output expressed as a fraction of the rate of energy output (heat rate + power) during a small number of cycles of sinusoidal movement with stimulation in part of each cycle (Curtin and Woledge 1993a, b).

The experiments on spinal catshark referred to in the Introduction showed that red fibres are active during slow swimming movements and whites during faster movements (Bone 1966). This pattern has been observed in many other species of fish ranging from eels through fish that use modes of swimming in which propulsion depends strongly on the movements of the tail and the body shape remains more streamline than in the swimming catshark (for example, skipjack tuna (*Katsuwonus pelamis*), Rayner and Keenan 1967; scup (*Stenotomus chrysops*) Rome et al 1993; bluegill sunfish (*Lepomis macrochirus*) Jayne and Lauder 1994; American eel (*Anguilla rostrata*) Gillis 1998).

### Fast swimming

In the current study the catshark chose their swimming speed and fast swims were relatively infrequent, about 15% of all the swims we observed. The data points we have (Fig. 3a, b) suggest that increasing fast swim speed is achieved by increasing stride-frequency, rather than stride-length. It is interesting that these relationships are different than those during slow swims (Fig. 3a, b). Additional evidence about fast swims would be beneficial for at least two reasons. First, there are very few data points for the faster swims (Fig. 3). Second, it is an open question where the swim speeds we observed are in relation to the maximum speeds the catshark can achieve when motivated. The lack of evidence about maximum swim speeds fits with the description (Butler et al 1986) of the catshark being unsuitable for study in a water channel, in which other species can be trained to increase swim speed as water flow increases. Instead, catsharks ‘merely angled their pectoral fins so that the water flowing over them generated a downward force, thus enabling the fish to remain motionless on the bottom’ (Butler et al. 1986).

Comparing the fast swims observed here with the results of in vitro experiments shows that the white fibres performed better at frequencies above the range we observed during the fast swims. The white fibres were most efficient at frequencies between 2.0 and 2.5 s^−1^ and produced their highest power at frequencies between 3.3 and 4.0 s^−1^, whereas the stride-frequencies for fast swims were lower, between 0.91 and 1.4 s^−1^ (values from the fitted line in Fig. 3b for fast swims). Thus, evidence from in vitro experiments on white fibres suggests that they do have the contractile capacity to provide the main propulsion for faster swimming than we observed here. Further evidence on this point is needed.

### Slow swimming

Most of the swims we observed here were in the slow group (Fig. 2). For this group our results show that stride-length increased strongly as swimming speed increased, whereas the stride-frequency did not increase much as swimming speed increased. The stride-frequency in catshark, corresponds to the frequency of the undulation of the fish body from the snout along the body and the tail (Gray 1933).

In the group of slow swims we observed, the range of speeds was 0.258–0.534 FL s^−1^, which correspond to frequencies 0.724–0.860 s^−1^, and the median speed, 0.397 FL s^−1^ corresponds to stride-frequency 0.783 s^−1^ (frequencies calculated from the fitted line in Fig. 3b). The histogram in Fig. 4b shows that the two most highly populated bins centred on frequencies 0.70 and 0.75 s^−1^ and contained values from 61% of the slow swims in which stride-frequency could be measured (70/115).

How do these preferred frequencies for slow swimming compare with the results of the experiments on red fibres in vitro? As indicated above we used the work-loop technique in our in vitro experiments to evaluate power and efficiency. At each frequency of movement, the timing of electrical stimulation was adjusted to optimize power and efficiency. Donley and Shadwick (2003) found with EMG and sonomicrometry recordings from red fibres in swimming leopard sharks that the pattern of activation relative to the movement cycle did not vary along the length of the body. Thus, our results which were from fibre bundles from a single location, likely apply along the length of the body.

**Fig. 5:**
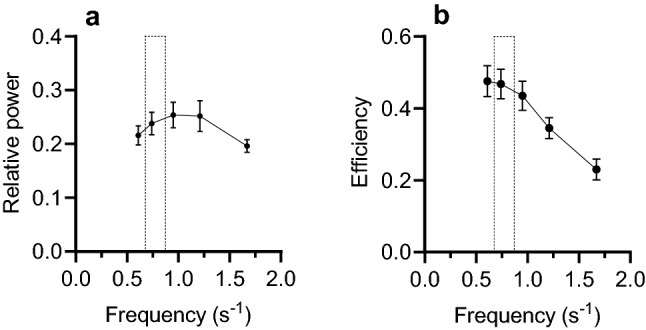
Comparison of the in vivo slow swims and in vitro red fibre energetics. The in vivo stride-frequency range (0.68–0.88 s^−1^) during slow swims reported here are shown as vertical bars in *Panels a* and *b*. The bars includes 61% of the slow swims (70/115). The in vitro dependence of **a** power and **b** efficiency on the frequency of sinusoidal movement by red fibres from catshark. Stimulation lasted for 33% of the movement cycle. The phase of the stimulation (start time during the movement cycle) was optimized for efficiency. Power and efficiency for these conditions is shown (mean values ± 1 SEM, results for 9 fibres bundles) (Curtin & Woledge 1993a, b)

Figure 5a and b show how the power and efficiency of red fibres varied with frequency of sinusoidal movement (Curtin and Woledge 1993a, b). The vertical bars indicate the range of stride-frequencies (frequency of the body movements) observed during slow swimming. There is a good match between this narrow range of frequencies observed during swimming and the frequencies that gave high performance by red fibres in in vitro experiments. Figure 5a shows that lower frequencies would give less power and Fig. 5b shows that higher frequencies lower efficiency. We conclude that the catshark uses the narrow range of stride-frequencies during slow swimming to get the best combination of power and efficiency from its red fibres. The narrow range of stride-frequencies requires that the increase in speed during slow swimming must be largely driven by the increase in stride length due to the undulating body pushing more forcefully against the water. In terms of muscle function, the increase in force is most likely due to recruitment (activation) of more muscle fibres to contribute to active force production.

### Supplementary Information

Below is the link to the electronic supplementary material.

Supplementary file1 (XLSX 22 KB)

## Data Availability

The summary data that support the findings in this study are provided in the supplementary material. Other data from the tracking analysis are available from the corresponding author on reasonable request.
